# Bioinformatic Analysis of Genome-Predicted Bat Cathelicidins

**DOI:** 10.3390/molecules26061811

**Published:** 2021-03-23

**Authors:** José Manuel Pérez de la Lastra, Patricia Asensio-Calavia, Sergio González-Acosta, Victoria Baca-González, Antonio Morales-delaNuez

**Affiliations:** 1 Biotechnology of Macromolecules Research Group, Instituto de Productos Naturales y Agrobiología, (IPNA-CSIC), Avda. Astrofísico Francisco Sánchez, 3, 38206 San Cristóbal de la Laguna, Spain; sergi_glez@hotmail.com (S.G.-A.); victoria@ipna.csic.es (V.B.-G.); morales.delanuez@ipna.csic.es (A.M.-d.); 2 Biological Activity Service, Instituto de Productos Naturales y Agrobiología, (IPNA-CSIC), Avda. Astrofísico Francisco Sánchez, 3, 38206 San Cristóbal de la Laguna, Spain; patriciaac@ipna.csic.es

**Keywords:** cathelicidin, bat, bioinformatics, genome, in silico, SARS-COV-2

## Abstract

Bats are unique in their potential to serve as reservoir hosts for intracellular pathogens. Recently, the impact of COVID-19 has relegated bats from biomedical darkness to the frontline of public health as bats are the natural reservoir of many viruses, including SARS-Cov-2. Many bat genomes have been sequenced recently, and sequences coding for antimicrobial peptides are available in the public databases. Here we provide a structural analysis of genome-predicted bat cathelicidins as components of their innate immunity. A total of 32 unique protein sequences were retrieved from the NCBI database. Interestingly, some bat species contained more than one cathelicidin. We examined the conserved cysteines within the cathelin-like domain and the peptide portion of each sequence and revealed phylogenetic relationships and structural dissimilarities. The antibacterial, antifungal, and antiviral activity of peptides was examined using bioinformatic tools. The peptides were modeled and subjected to docking analysis with the region binding domain (RBD) region of the SARS-CoV-2 Spike protein. The appearance of multiple forms of cathelicidins verifies the complex microbial challenges encountered by these species. Learning more about antiviral defenses of bats and how they drive virus evolution will help scientists to investigate the function of antimicrobial peptides in these species.

## 1. Introduction

The planet is facing a global viral pandemic with catastrophic implications for human life and socio-economic activity. This pandemic is triggered by a zoonotic coronavirus, SARS-CoV-2, which is presumed to occur in bats and could have been transmitted to humans by an intermediary host animal. Coronaviruses (CoVs) are RNA viruses that cause respiratory and enteric diseases with varying pathogenicity in humans and animals. All CoVs considered to infect human beings are zoonotic or animal in nature, many assumed to be from bat hosts [1]. While bats have a long history of association with rabies, the appearance and re-emergence of a variety of bat viruses, such as coronavirus SARS-CoV-2, that have an impact on human and animal health has resulted in a re-emergence of interest in bat immunology. It was proposed that one of the natural reservoirs of the SARS-CoVs could be the horseshoe bat (genus *Rhinolophus*) [2].

Bats (Order *Chiroptera*) are a particularly striking group of mammals; indeed, they appear in many legends, myths, and fables. They are the only mammals capable of active flight, like birds. However, unlike most birds, bats are active at night, or at least at sunset and sunrise. This behavior, which allows them to avoid ecological competition with birds, has been possible thanks to the development of echolocation systems that allows them to fly even in complete darkness. Bats are the second order of mammals in number of species, only surpassed by rodents [3]. At the risk of certain drawbacks, bats provide humans with certain clear benefits. Their dung has been mined for use as fertilizer. Insectivorous bats are inherently effective controllers of pests, reducing the need for pesticides. However, fruit bats are often considered pests by fruit growers. Bats are the only mammals capable of prolonged flight and are considered to be the carriers of some of the most extremely pathogenic viruses in the world, including Nipah, Hendra, Ebola, and extreme acute respiratory syndrome (SARS) [4]. Bats are capable of coexisting with viruses and may have established strategies to regulate viral replication more successfully than most other mammals [5]. Data from laboratory and naturally infected bats have shown that they rarely have pathological signs, while they can be persistently infected with certain viruses [6]. Being the second most species-rich and widespread order of mammals, bats are still among the least studied, with a specific lack of knowledge in the field of bat immunology. Like other species, their immune systems contain innate and adaptive functions [7]. In recent years, attempts to understand these systems have been significantly facilitated by the availability of genome sequences. Bat1K (https://bat1k.ucd.ie, accessed on: 10 January 2021) is an initiative to sequence the genomes of all their living species, approximately 1300 in total. The main goal of this consortium is to uncover the genes and genetic mechanisms behind the unusual adaptations of bats, essentially to mine their genomes to uncover the secrets of longer health-spans, flight, echolocation, and disease resistance. [8].

Genetically encoded antimicrobial peptides (AMPs) have been found to be essential elements in reaction to epithelial compromise and microbial invasion. Antimicrobial peptides play a crucial role in ensuring innate immunity before activating the adaptive immune response in higher organisms, which requires the development of antibodies and specialized cells. Several such peptides, including well-characterized defensins and cathelicidins, have been identified in mammals. Increased production of beta defensin 103 and hepcidin is recorded in many bat tissues compared to humans [9]. This leads to the assumption that bats are more resistant to infection due to over-expression of these antimicrobial peptides.

Cathelicidins is the second largest class of antimicrobial peptides in higher eukaryotes. These cationic peptides inactivate microbes by a general process, ideally through attaching to their membranes and inducing membrane disruption [10]. Cathelicidins are characterized as antimicrobial peptides derived from pre-propeptide proteolytic cleavage, which includes the domain of cathelin in mammals and the less well-preserved cathelin domain in other eukaryotes. In humans, cathelicidins are retained as inactive prepropeptides in azurophilic neutrophil granules and are converted by enzymes (neutrophil elastase or serine protease) into a mature active peptide [11]. In humans and higher vertebrates, the active cathelicidin peptide is always encoded by exon 4 of the cathelicidin encoding gene [9]. A biochemical relation between vitamin D, LL-37 and COVID-19 intensity has been suggested for the human cathelicidin LL-37. Surface Plasmon Resonance research has shown that LL-37 binds to SARS-CoV-2 S protein and prevents binding to its hACE2 receptor, and most likely viral entry into the cell [12].

We examine in this report the predicted cathelicidin antimicrobial peptides of bats and provide some structural, phylogenetic, and in silico analysis of the predicted peptides. The study of how these interesting creatures use antimicrobial peptides within their innate immune systems may reveal new insights into our innate immune system and also provide new and powerful antimicrobial peptides for potential therapeutic development.

## 2. Results

To analyze the number of cathelicidins in each bat species, we searched the NCBI database. A total of 32 unique proteins were retrieved (Table 1). Interestingly, some bat species contained more than one cathelicidin. For example, *Myotis lucifugus* and *Phyllostomus discolor* comprised four sequences; *Myotis myotis*, and *Rhinolophus ferrumequinum* contained three unique annotated cathelicidins, whereas some bat species; such as *Artibeus jamaicensis*, *Eptesicus fuscus*, *Hipposideros armiger*, *Miniopterus natalensis*, *Molossus molossus*, *Pipistrellus kuhlii*, *Pteropus alecto*, and *Pteropus vampyrus* contained a single sequence. To date, several bat genomes are being sequenced and submitted to the NCBI database. To ascertain whether the number of cathelicidins found in bat species was somehow related to their available genome sequences, we searched the NCBI for the corresponding genome project and compared with the median genome length and protein counts. We could see that for the genome of *Molossus molossus*, with a median length of 2315 Mb and 53,797 protein counts, there is only one cathelicidin, whereas for other bat genomes; such as *Myotis lucifigus*, with a median length of 2034 Mb and 43,106 protein counts, we could find four distinct cathelicidins.

The 32 unique bat cathelicidin sequences were retrieved from the NCBI database in FASTA format (Supplementary Material Figure S1) and subjected to further structural analysis.

Cathelicidins are a particularly unusual class of peptides since their primary structure consists of a closely conserved propeptide domain, where the sequence of the mature peptide may be greatly divergent. They are characterized by the existence of an N-terminal signal sequence, a conserved cathelin-like domain, and a variable C-terminal domain, which becomes a mature, proteolytic, functional peptide. Cathelin-like domains have high-sequence homology to cathelin, a porcine leukocyte protein belonging to the cystatin class of cysteine protease inhibitors. We first aimed to identify the conserved cathelin-like domain within the sequences, using the “batch conserved domain” search of the NCBI (https://www.ncbi.nlm.nih.gov/Structure/bwrpsb/bwrpsb.cgi, accessed on: 10 January 2021) and its alignment with the superfamily member pfam00666. The information obtained allowed us to identify the conserved region of the pre-propeptide and separate it from the putative active portion of the cathelicidin peptide. However, we detected some incomplete bat cathelicidin sequence; sometimes because they lacked the four cysteine residues typical of the cathelin domain, in others because the length of the active cathelicidin peptide was very short to be considered a true peptide (Table 2). Therefore, these sequences were not subjected to further structural analysis.

**Figure 1:**
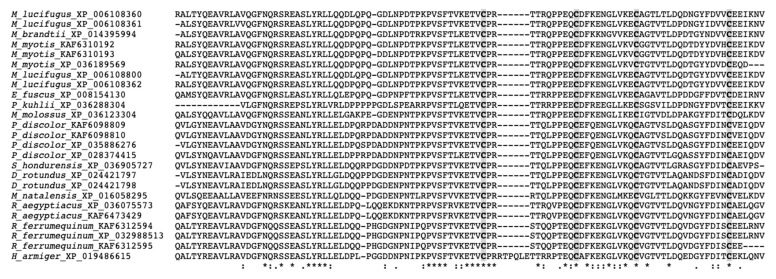
Multi-sequence alignments of the conserved cathelin region of bat cathelicidins using the default parameters of CLUSTALW (https://www.genome.jp/tools-bin/clustalw, accessed on: 10 January 2021). Gaps are inserted to optimize the alignment. Conserved cysteines are shaded and identical amino acids are indicated by asterisks, whereas those with high or low similarities are indicated by colons and dots, respectively.

We next used the conserved cathelin domain to align the region and determine possible phylogenetic relationships using this domain. Multi-sequence alignments of bat cathelicidins were conducted on a pro-region basis (Figure 1). Noticeably, four cysteines that were conserved in the cathelin domain of all bat cathelicidin precursors were invariantly spaced, except the sequence of *Hipposideros armiger*. In this case, the sequence was longer than the rest of the sequences and included the additional residues RTPQLE, which lie between the first and second cysteines of the cathelin-like domain (Figure 1).

**Figure 2:**
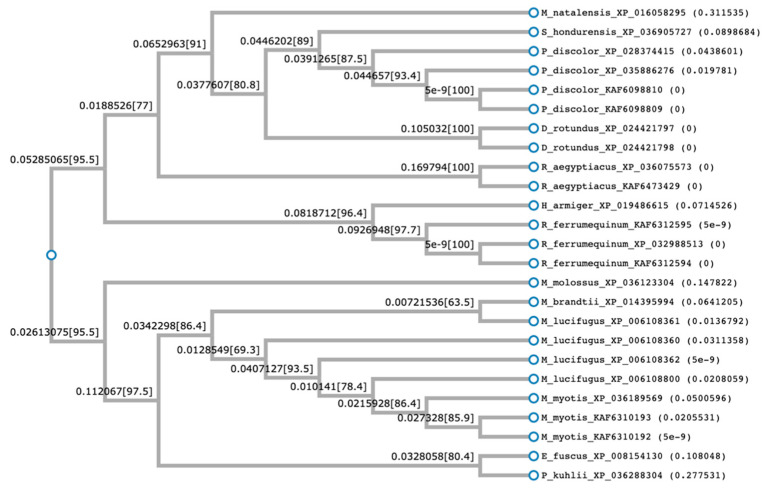
Phylogenetic tree constructed from the multi-sequence alignments of the conserved cathelin region of bat cathelicidins. The tree was constructed using FastTree with default parameters. FastTree infers maximum-likelihood phylogenetic trees using the Neighbor Joining method and computes local support values with the Shimodaira-Hasegawa test.

Using this alignment of cathelin-like domains, a phylogenetic tree was built (Figure 2). Phylogenetic analysis of the cathelin-like segment of bat sequences revealed significant similarity of most bat sequences. Interestingly, the sequences fall into two branches. The upper branch consists of two clusters: one represented by the sequence from the bats *M. natalensis*, *S. hondurensis*, *P. discolor*, *D. rotundus*, and *R. aegyptiacus*. The other cluster represented by the sequences of *H. armiger* and *R. ferrumequinum*. The lower branch was represented by the sequences from all genus *Myotis* (*M. lucifugus*, *M. davidii* and two *M. brandtii* sequences) in a consistent cluster. Located in the second cluster are the sequences from *E. fuscus* and *P. kuhlii*. The sequence from *M. molossus* was separated but still related to the lower branch of the tree. This suggests that cathelicidins in this bat-specific cluster arose earlier than the other species, although from a common ancestor (Figure 2).

### 2.1. Prediction of Cathelicidin Active Peptides

We noticed that most cathelin-like regions predicted for the bat cathelicidin sequences ended in a valine or alanine. In most of the cathelicidins described so far, processing of their precursors into mature antimicrobial peptides is regulated by elastase [13]. The elastase-sensitive residue is usually a valine or an alanine located near the start of exon IV in the elastase-processed cathelicidins. Therefore, we considered the active cathelicidin peptide as the C-terminal remaining portion of the bat sequences after removal of their cathelin-like region defined by the motif pfam00666 (Table 3). The length of the peptides ranged between 30 and 50 amino acids. However, two peptides; corresponding to bat sequences XP_036189569 and XP_036288304 from *M. myotis* and *P. kuhlii*, respectively were unusually long, containing more than 85 residues. We also noticed that most peptides contained the LPS binding domain of CAP18 (C-terminal). This domain was detected with the alignment with the superfamily member pfam12153, which is found in most cathelicidin in association with pfam00666, and is approximately 30 amino acids in length. CAP18 is a protein which is derived from rabbit granulocytes that contains a C-terminal gram-negative LPS binding domain (Table 3).

Interestingly, some cathelicidins such as XP_035886276 and XP_028374415 (*P. discolor*), or KAF6310193 and KAF6310192 (*M. myotis*) contained the same active peptide (Table 3) despite having distinct cathelin-like regions (Table 1). Therefore, after removing duplicates sequences, a total of 20 different antimicrobial peptides were finally considered for the in silico predictions of structure and activity (Table 3).

### 2.2. Structural and Functional Prediction of Bat Cathelicidins

**Figure 3:**
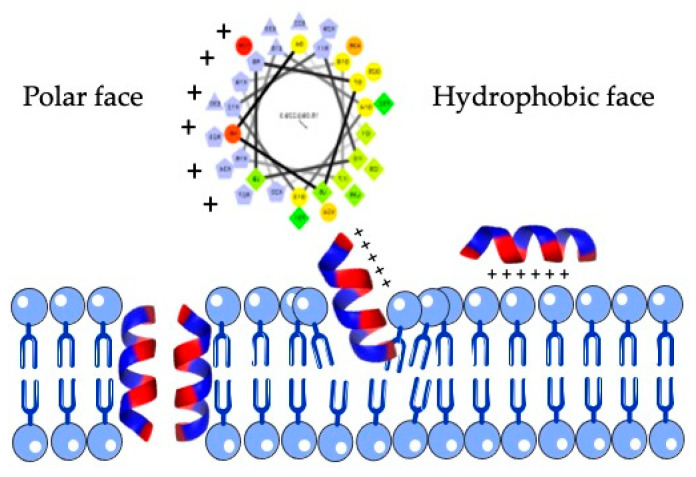
Structure of an amphipathic helix. Yellow/green residues of the upper helical view are hydrophobic and form the hydrophobic face, whereas purple residues of the helix are polar residues and are located at the opposite face. This feature is typical of most antimicrobial peptides and allow them to interact with membranes (down).

The cathelicidin family of host defense peptides comprise a group of cationic and typically amphipathic peptides that show a range of activities correlated with host defense functions, including antimicrobial activities. The resulting mature cathelicidin peptides are considerably varied in length, amino acid sequence, and shape; differing in their alpha-helical, elongated or beta-hairpin configurations. As an internal control of the bioinfomatic analysis, the human cathelicidin peptide LL-37 was included (Table 3). Most cathelicidins are often characterized by having an amphipathic helix, with one face made of hydrophobic residues inserted between the lipid acyl chains and the opposite face containing polar residues that allow them to interact with membranes (Figure 3).

**Figure 4:**
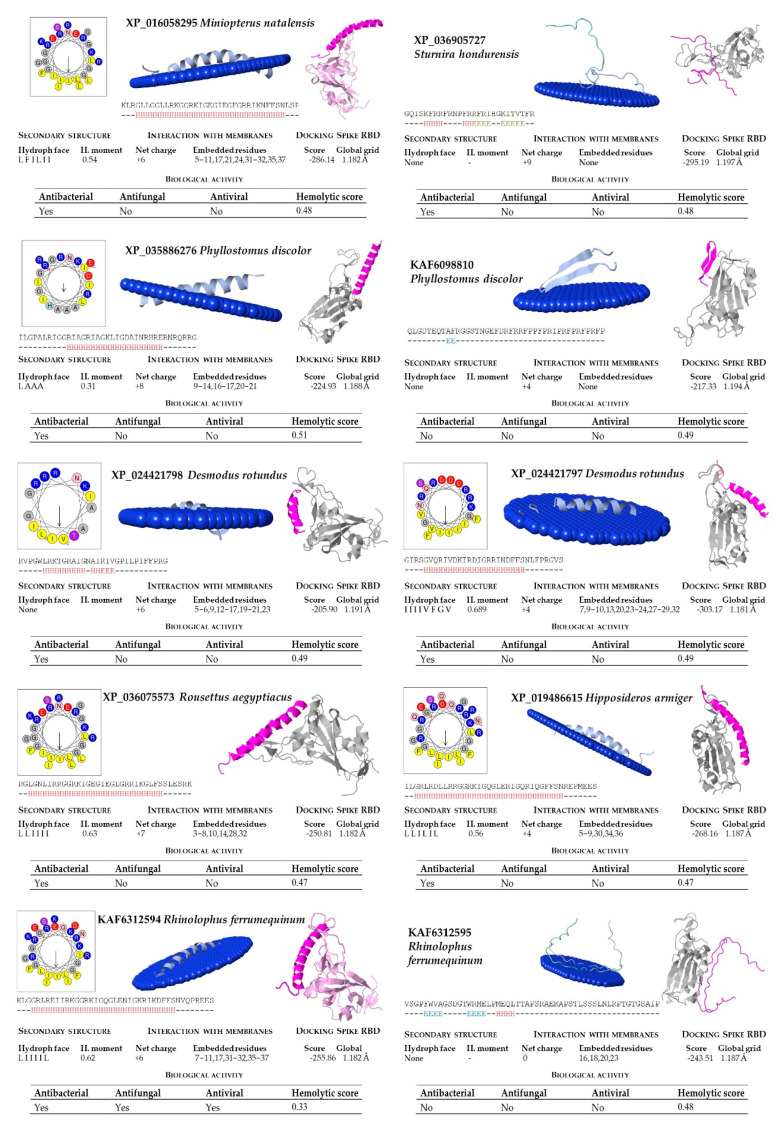
Bioinformatic analysis of bat cathelicidins (part 1). Net charge, secondary structure, hydrophobicity of helixes, and interaction with membranes and SARS-CoV-2 region binding domain (RBD) of the Spike protein were computed. The analysis includes predictions of biological properties of the peptides, such as antibacterial, antifungal, antiviral, and hemolytic activities.

**Figure 5:**
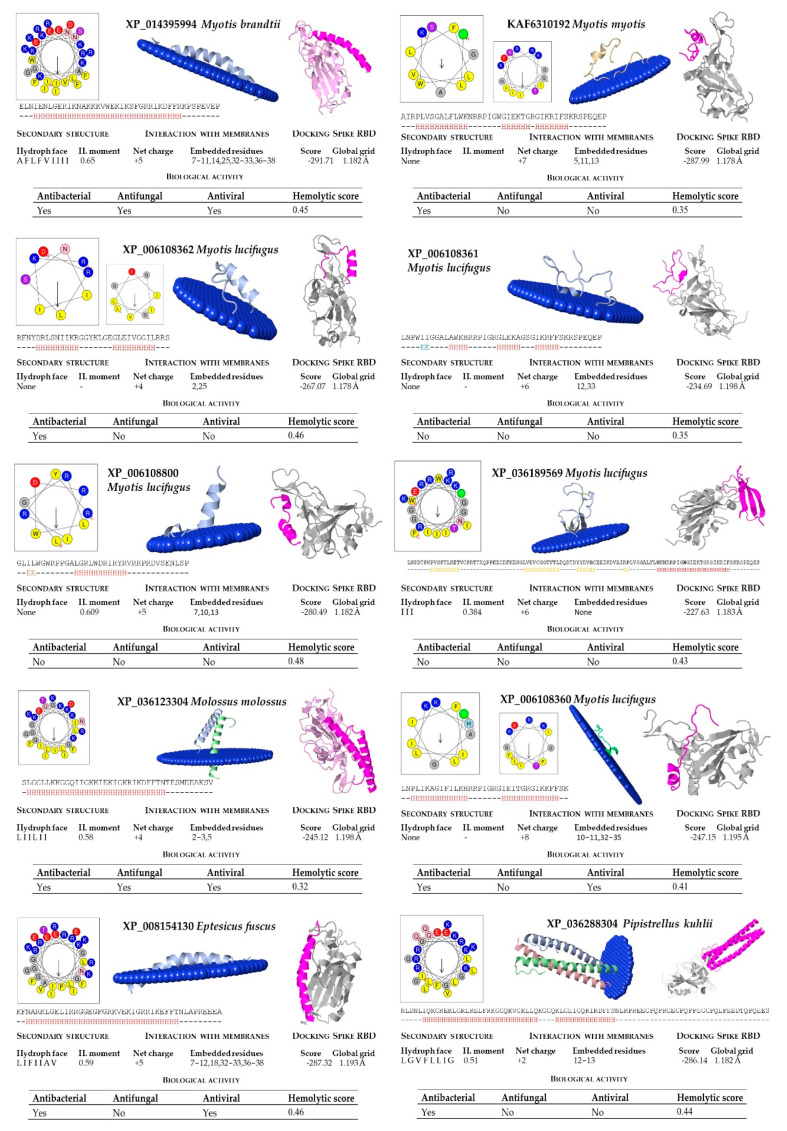
Bioinformatic analysis of bat cathelicidins (part 2). Net charge, secondary structure, hydrophobicity of helixes, interaction with membranes and with the SARS-CoV-2 region binding domain (RBD) of the Spike protein were computed. The analysis included predictions of biological properties of the peptides, such as antibacterial, antifungal, antiviral, and hemolytic activities.

**Figure 6:**
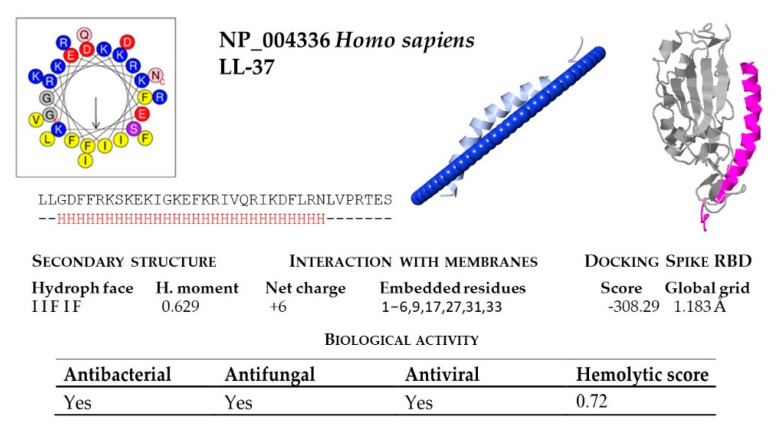
Bioinformatic analysis of human cathelicidin peptide LL-37. Net charge, secondary structure, hydrophobicity of helixes, interaction with membranes and with the SARS-CoV-2 region binding domain (RBD) of the Spike protein were computed. The analysis included predictions of biological properties of the peptides; such as antibacterial, antifungal, antiviral and hemolytic activities.

In our structural analysis, we first computed the net charge of the peptides and the secondary structure, determined by the hydrogen bonding pattern. All the peptide sequences, except KAF6312595 from *R. ferrumequinum*, were recognized as cationics, with net charges ranging between +2 (from *P. kuhlii*) and +9 (*S. hondurensis*) (Figure 4 and Figure 5). According to the analysis of the secondary structure, most bat cathelicidins were predicted to have alpha helixes (Figure 4 and Figure 5). To study their amphipathic character of these helixes, we used the helical wheel projection tool and computed the hydrophobic moment. The sequences from *P. discolor* (KAF6098810), *R. ferrumequinum* (KAF6312595), *S. hondurensis* (XP_036905727) and *M. lucifugus* (XP_006108361) did not have helical content in at least eight contiguous residues and this parameter was not computed. A large number of servers and tools are dedicated to predicting the biological activity of antimicrobial peptides. To predict the biological activity of the peptides, we computed the antibacterial, antifungal, antiviral, and hemolytic activities of the bat cathelicidins (Figure 4 and Figure 5). Single antibacterial activity was predicted for 10 of the peptides, whereas three peptides: XP_014395994 (*M. brandtii*), XP_036123304 (*M. molossus*) and KAF6312594 (*R. ferrumequinum*) were predicted to have antibacterial, antifungal, and antiviral activity, consistent with the human cathelicidin LL-37 (Figure 6). A total of five peptides did not exhibit any activity with our prediction methods.

The hemolytic activity of peptides is an indicator of their toxicity in eukaryotic cells, and is a characteristic widely determined for compounds that may come into contact with the human body. All peptides subjected to in silico analysis showed no hemolytic activity using the HAPPENN server. Using the HemoPI server the hemolytic score (probability from 0-no hemolitic to 1-hemolitic) of the peptides ranged between 0.32 for XP_036123304 (*M. molossus*) and 0.51 for XP_035886276 (*P. discolor*). The highest value of 0.72 was obtained for the human LL-37 (Figure 6).

The diversity of cathelicidin peptides accounts for their distinct functions and diverse range of action and/or antimicrobial potency. We next performed biophysical and structural studies with the tertiary structure of the predicted bat sequences to explore the mechanisms by which the significant bat peptides interact with simulated membranes and other proteins. For that purpose, we modeled every peptide and subjected them to an in silico study of this interaction. We found that all of these peptides potentially interacted with membranes, with the exception of KAF6098810 (*P. discolor*) and XP_036905727 (*S. hondurensis*) (Figure 4 and Figure 5). The peptides from *R. ferrumequinum* (KAF6312595), *M. lucifugus* (XP_006108361, XP_006108362), *M. myotis* (KAF6310193), *M. molossus* (KAF6430041), and *P. kuhlii* (KAF6334921) showed a weak interaction with membranes, with only 2–4 residues embedded in the simulated membrane.

Human cathelicidin LL-37 was recently reported to bind to the region binding domain (RBD) of the Spike protein of SARS-CoV-2 coronavirus [12]. Using the COVID-19 docking server, we submitted the bat cathelicidin peptide models to an analysis of their interaction with the RBD domain of the SARS-CoV-2 Spike protein. In general, the score value of the interactions ranged between −200 and −300 (Figure 4 and Figure 5). For some peptides, such as KAF6098810 and XP_035886276 (*P. discolor)*, KAF6312595 (*R. ferrumequinum*), XP_006108361 (*Myotis lucifugus*), KAF6310193 (*M. myotis*), KAF6430041 (*M. molossus*), XP_024421798 (*D. rotundus*), and KAF6334921 (*P. kuhlii*), the score of this interaction was below −250. The lowest score of −303.17 was obtained for *D. rotundus* cathelicidin (XP_024421797). As a reference, the score for the human LL-37 was −308.29 (Figure 6).

## 3. Discussion

It has been argued that the immense diversity of cationic peptides arises from their antimicrobial function to fight against the range of pathogenic microbe challenges they face in each host organism. We consider that nature still harbors a virtually infinite array of potential peptide medications that await human pharmacological characterization. In particular, organisms living in germ-filled environments are an abundant source of antimicrobials that can likely provide us with superior templates for use in developing new antimicrobial drugs to help solve the daunting medical problems that still persist today [14].

We previously described the characterization of peptides derived from the genomes of marine, flying, and terrestrial mammals [15]. Bats are exceptionally long-lived, questioning the reported favorable association between body mass and overall life span. They are the only mammals that have attained powered flight and the only non-marine vertebrates that echolocate. Bats are outstanding in their potential to serve as reservoir hosts for intracellular pathogens. Despite increasing understanding of bats’ role in environmental and human health and disease, we know very little regarding how they deal with their parasites, viruses, and commensal microbes [8]. Certain animals have a single cathelicidin family member (such as wolves, rodents, rats, and humans), whereas others have five or more distinct members [16]. In our study, some bat genomes such as *M. brandtii* exhibited up to four cathelicidins, whereas others such as *M. molossus* appear to have only one. There are some unknowns about the origin and phylogeny of bats [17]. Two large sub-orders were considered, the *Microchiroptera* and *Megachiroptera*. The first are insectivorous or secondarily adapted to other diets, such as blood (Vampire bat) or fruit (Flying fox). Phylogenetic research into chicken cathelicidins, for instance, showed that genes encoding cathelicidins and mammalian neutrophilic granule peptides are presumably descendants of a single, remotely linked gene that developed prior to the separation of birds and mammals. However, it is thought that genes encoding other mammalian cathelicidins may have been duplicated from the ancestral neutrophilic granule peptide gene after mammals and birds drifted apart [13,18,19]. Consistent with our phylogenetic analysis of the cathelin-like domain, the bat species *M. molossus*, containing a single cathelicidin, seems to have arisen earlier from a common ancestor with other species.

The cathelicidin family of endogenous antimicrobial peptides plays a vital function in the mammalian innate immune response against bacterial infection. The potential of cathelicidin-derived antimicrobial peptides has been recognized, owing to their greater bactericidal activity compared to chemical drugs, as well as their remarkable mode of action, which hinders development of drug resistance [20].

All cathelicidins are encoded by genes with four exons. The first exon encodes a signal peptide of 29–30 residues, while exons 2 and 3 encode the cathelin domain of 99–114 aa. Exon 4 encodes a mature peptide with an antimicrobial domain of 12–100 aa. Among species and numerous peptides, the N-terminal signal sequence and pro-region (cathelin) are strongly conserved, whereas the C-terminal domain encoding the mature peptide displays considerable heterogeneity [21].

This study was aimed at the identification and characterization of bat cathelicidins using the NCBI genome database. The annotation of the sequences as bat cathelicidins is an automated computational process carried out by the NCBI using a gene prediction method called Gnomon. The nature of the cathelicidin family was established based on the presence of a conserved cathelin-like domain. We examined the expected antimicrobial peptides of bat cathelicidins and provide some structural and bioinformatics-based evidence to confirm their antimicrobial action. In our analysis of 32 unique bat cathelicidin sequences, we had to reject a total of seven sequences due to lack of conservation of four cysteines and/or incomplete cathelin-like portions. Moreover, two cathelicidins seemed to lack the sequence coding for the active mature antimicrobial peptide, whereas other exhibited unusually long forms of the antimicrobial peptide. This raises questions about the automated computational analysis of cathelicidins using this gene prediction method and protein evidence is necessary to confirm the unusual version of these predicted sequences.

Proteolytic maturation is essential for cathelicidin to perform its antimicrobial action. It is widely accepted that the molecule is elastase-processed in fish, birds, and mammals [22]. The removal of the cathelin-like domain yields the active antimicrobial peptide. In the case of mammalian sequences, valine is the most abundant residue susceptible to elastase. After removal of duplicates, we ended up with 20 putative active peptide sequences corresponding to 25 bat cathelicidins. In vivo, cationic antimicrobial peptides are targeted to the cell surface of several microorganisms through electrostatic interactions [23]. Cathelicidins show preference for negatively charged prokaryotic membranes with high electrical potential gradients, as a requirement for cell entry or direct destruction of the bacterial cell membrane [24,25]. This feature was computed using available bioinformatic tools that predicted the antibacterial, antifungal, and antiviral activities. In our study, we also modeled the predicted active peptides from bat cathelicidins and computed the interaction with simulated membranes. Interaction of cationic peptides and negatively charged lipid membranes of microorganisms allows precise, concurrent adhesion and anchoring that permits the disturbance of the microbial membrane. Changing the secondary and tertiary configuration of the peptide may shift its perpendicular direction, thereby embedding it into the lipid bilayer and forming transmembrane pores (Figure 3). There are many ways that peptides can interact with the cytoplasmic membrane. The “barrel stave” mechanism is based on the raising of structures that resemble the aligned wooden staves of a barrel, which include the hydrophilic inner surface of the “staves”. Another process is called “connecting channels”, by which proteins combine with the cell membrane and open pores into the cell [26,27,28]. Our in silico analysis was consistent with the net charge and secondary structure of each peptide, since those with lower net charge and/or lower helicity showed a lesser extent of interaction with simulated membranes, as revealed by the lower number of membrane-embedded residues. Consistent with our analysis, human cathelicidin LL-37 lies roughly parallel to the bilayer plane and does not penetrate deeply into the hydrophobic core of the membrane [29].

Since the original discovery of the lyssavirus in asymptomatic vampire bats in 1911, the link between bats and human disease has been recognized for over a century [30]. Until recently, rabies dominated the scientific literature on bats and diseases. Presently, bats are also linked with the production of many destructive infectious diseases in humans and other animals [31]. Interestingly, with the exception of rabies and other lyssaviruses, viruses do not tend to induce active pathology in bats, indicating the existence of benign interactions between bats and their pathogens. Novel viral diseases, such as COVID-19, create real challenges to human health and economic development. It is, therefore, important to better understand the existence and diversity of pathways responsible for reservoir hosts’ ability to survive viral infections, and promote their spread to other host organisms [32]. Viruses only disperse within living cells, and their lifecycles comprise five stages: host cell connection, fusion/cell entry, host cell multiplication, host cell assembly, and host cell release [31]. Usually, antiviral peptides attack structures that are important for viral replication. For example, they can attack viral proteins and interfere with their interactions, alter enzymatic behavior, modulate conformation changes, or impact host proteins that are necessary for virus infection, such as viral proteins triggering entry receptors or proteases. There may be a specific equilibrium between host protection and immune tolerance in bats, and this particular balance may be responsible for the special relationship between bats and viruses (particularly coronaviruses). Recently, the human cathelicidin LL-37 has been reported to bind to the RBD of SARS-CoV-2 [33]. Therefore, we wondered whether cathelicidins might be involved in the protection from coronavirus in bat species. We modeled corresponding bat cathelicidin peptides and tried to dock the molecules to ascertain if they also interact with this region of the S protein. For some species, such as the vampire bat *D. rotundus*, we found similar scores as the one found for human cathelicidin. However, our docking analysis is not conclusive, as peptides that target domains other than RBD in the S protein can also interfere with viral entry. Because there is no bat animal model, we have minimal resources and reagents. Therefore, our knowledge about the function of their immune responses in the regulation of viral infections is limited [17]. It is thought that the various dampening processes in bats, which in turn reduce excessive virus-induced or age-related inflammation, may have been induced by flight adaptations, which may ultimately lead to virus tolerance and increased bat longevity [34]. Understanding how bats coexist with viruses in the absence of illness is important in the designing of therapies to target viruses in humans, vulnerable livestock, and pet animals [35]. Such work is important to understand the immune responses that dictate the relationship between virus persistence and immunopathology in reservoir hosts and may help in elucidating human viral pathogenesis and reveal possible targets for therapeutic intervention [36]. This bioinformatic study of bat cathelicidins could provide scientists with several avenues to identify the functions of antimicrobial peptides in these species.

## 4. Materials and Methods

### 4.1. Retrieval of Bat Cathelicidin Sequences

Bat genome sequencing projects were examined (https://bat1k.ucd.ie, https://www.ncbi.nlm.nih.gov/genome, accessed on: 10 January 2021) and cathelicidins were searched in the in Identical Protein Groups database of the NCBI (https://www.ncbi.nlm.nih.gov/ipg/docs/about, accessed on: 10 January 2021) by entering the words “cathelicidin” or “cathelin-like” and “chiroptera”. The sequences were downloaded in FASTA format for further analysis.

### 4.2. Structural Analysis

To elucidate protein function, bat sequences were submitted to a search for conserved domains using the CD-Search service [37] (https://www.ncbi.nlm.nih.gov/Structure/bwrpsb/bwrpsb.cgi, accessed on: 10 January 2021). This server uses “reverse position-specific BLAST” to compare a query protein sequence against conserved domain models collected from several source databases, and presents the results as a concise display. If CD-Search finds a specific hit, there is high confidence in the association between the protein query sequence and a conserved domain, also resulting in a high confidence level for the inferred function of the protein query sequence.

### 4.3. Multiple Alignment and Phylogenetic Tree

We performed a multiple alignment of bat cathelicidin proteins using the default parameters of CLUSTALW tool (https://www.genome.jp/tools-bin/clustalw, accessed on: 10 January 2021). Alignment and phylogenetic reconstructions were carried out with the function “build” of ETE3 v3.1.1 [38], as implemented in GenomeNet (https://www.genome.jp/tools/ete/, accessed on: 10 January 2021). The tree was constructed using FastTree v2.1.8 with default parameters. FastTree computes local support values with the Shimodaira-Hasegawa (SH) test [39].

### 4.4. In silico Analysis of Biological Activity

To predict the hemolytic activity of the peptides, we applied HAPPENN (https://research.timmons.eu/happenn, accessed on: 10 January 2021), a neural network model trained with hemolytic and non-hemolytic peptides, both experimentally validated [40] and calculated the hemolytic score with the HemoPI server (https://webs.iiitd.edu.in/raghava/hemopi/batch.php, accessed on: 10 January 2021) that comprises of 552 experimentally validated highly hemolytic peptide sequences from Hemolytik Database as positive dataset and same number of peptide sequences randomly generated from SwissProt as negative dataset [41]. Antiviral properties of peptides were predicted by means of AVPpred (http://crdd.osdd.net/servers/avppred/index.html, accessed on: 10 January 2021), which uses an antiviral peptides prediction algorithm developed using peptides with experimentally proven antiviral activity. For prediction of antibacterial and antifungal peptides we employed the web server iAMP-2L (http://www.jci-bioinfo.cn/iAMP-2L, accessed on: 10 January 2021), which has an anticipated overall success rate over 86% [42].

### 4.5. Structural Analysis of Peptides

Helicity of the peptides was computed and visualized by the server HeliQuest (https://heliquest.ipmc.cnrs.fr/, accessed on: 10 January 2021). The hydrophobic moment <µH> ranging from to 0 to 3.26 was also calculated by this server. A large <µH> value means that the helix is amphipathic perpendicular to its axis.

We used JPred4 (http://www.compbio.dundee.ac.uk/jpred4/index.html, accessed on: 10 January 2021) for predicting secondary structures of peptides, classifying each amino acid residue as belonging to alpha helix (‘H’), beta sheet (‘E’), or not H or E (‘-’) secondary structures.

### 4.6. Modeling of Peptides

Models of bat cathelicidin active peptides were computed by the SWISS-MODEL server homology modeling pipeline (https://swissmodel.expasy.org, accessed on: 10 January 2021). This relies on ProMod3, an in-house comparative modeling engine based on OpenStructure. For the interaction of peptides with membranes we used the PPM server (https://opm.phar.umich.edu/ppm_server, accessed on: 10 January 2021), which calculates rotational and translational positions of transmembrane and peripheral proteins in membranes using their 3D structure (PDB coordinate file) as input.

### 4.7. Molecular Docking studies

For peptide docking, CoDockPP (http://codockpp.schanglab.org.cn/, accessed on: 10 January 2021) was used as docking engine. CoDockPP program provides a multistage fast Fourier transform (FFT)-based strategy for both global docking and site-specific docking and shows higher success rates and much more hit counts than does ZDOCK in global docking predicting much more accurate binding modes than RosettaDock in site-specific docking [43]. The region binding domain (RBD) of the spike protein was selected as the protein target. All the parameters were set as default and docking was performed in a global base. The COVID-19 docking server (https://ncov.schanglab.org.cn/index.php, accessed on: 10 January 2021) provides a user-friendly interface and binding mode visualization for the results, which makes it a useful tool for drug discovery of COVID-19. The server only displays the top 10 models. The models can be visualized in 3D by JSmol. The spike protein was colored in gray, and the predicted structure of Top 1 was colored in pink. TIn the TOP 1 model of the corresponding peptide is colored in pink.

## 5. Conclusions

The appearance of cathelicidins across a range of organisms verifies their importance. Owing probably to the complex microbial challenges encountered by these species, their heterogeneity is a valuable resource in the production of novel therapeutics. Learning more about antiviral defenses of bats and how they drive virus evolution will help scientists develop better ways to anticipate, avoid or limit the spread of their viruses to humans. Our preliminary bioinformatic study indicates that cathelicidin peptides are expected to be antimicrobial in these complex flying mammals. By comparison with human LL-37, we provide evidence to confirm the antimicrobial action of the predicted bat cathelicidins. Study of how bats use antimicrobial peptides within their innate immune systems may reveal new insights into our innate immune system and that of other mammals. It could also provide new and powerful antimicrobial peptides for potential therapeutic development.

## Figures and Tables

**Table 1:** Number of predicted unique cathelicidin proteins found for each bat species that have on-going sequencing projects by February 2021, in comparison with the median genome length and protein counts. Source: NCBI database (genome).

Bat Species	Genome ID	Length (Mb)	Proteins	Cathelicidins
*Artibeus jamaicensis*	12026	2316.20	42,600	1
*Desmodus rotundus*	15041	2063.80	29,845	2
*Eptesicus fuscus*	11703	2026.63	49,822	1
*Hipposideros armiger*	15002	2236.58	45,831	1
*Miniopterus natalensis*	44094	1803.10	29,787	1
*Molossus molossus*	93829	2315.57	53,797	1
*Myotis brandtii*	18281	2107.24	40,808	2
*Myotis davidii*	14635	2059.80	33,106	2
*Myotis lucifugus*	614	2034.58	43,106	4
*Myotis myotis*	43810	2148.60	61,156	3
*Phyllostomus discolor*	75334	2080.20	46,999	4
*Pipistrellus kuhlii*	93828	1775.69	39,923	1
*Pteropus alecto*	12056	1985.96	39,693	1
*Pteropus vampyrus*	757	2198.28	43,630	1
*Rhinolophus ferrumequinum*	10960	2072.56	45,117	3
*Rousettus aegyptiacus*	7672	1904.62	61,105	2
*Sturnira hondurensis*	95481	2098.36	43,530	2

**Table 2:** Bat cathelicidin sequences rejected from further structural analysis, due to lack of conserved cysteines within the cathelin-like region or to the missing of the portion of the cathelicidin active peptide. The interval shows the region of homology with the superfamily member pfam00666.

Bat Species	Accession No.	Interval	Cysteines	Peptide Length
*A. jamaicensis*	XP_036984419	30–104	2	14
*S. hondurensis*	XP_036905728	30–127	4	10
*M. brandtii*	XP_005867268	33–131	4	3
*M. davidii*	ELK24988	39–82	3	43
*M. davidii*	ELK24989	32–126	4	7
*P. alecto*	XP_006914980	23–102	3	67
*P. vampyrus*	XP_011373748	23–94	2	64

**Table 3:** Predicted cathelicidin active peptides of the bat sequences corresponding to the C-terminal portion of the protein after removal of the cathelin-like region.

Bat Species	Accession	Sequence of Active Peptide
*D. rotundus*	XP_024421798	RVPGWLRKTGRAIGNAIRIVGPILPIFFPRG
*D. rotundus*	XP_024421797	GIRSGVQRIVDKIRDIGRRINDFFSNLFPRGVS
*E. fuscus*	XP_008154130	KFNARKLGELIRRGGEGFGRKVEKIGRRIKEFFTNLAPREEEA
*H. armiger*	XP_019486615	ILGRLRDLLRRGGRKIGQGLERIGQRIQGFFSNREPMEES
*M. brandtii*	XP_014395994	ELNIENLGERIKNAKKKVWEKIKSFGRRIKDFFRKPSPEVEP
*M. lucifugus*	XP_006108362	RFNYDRLSNIIKRGGYKLGEGLEIVGGILRRS
*M. lucifugus*	XP_006108800	GLILWGWRPPGALGRLWDRIRYRVRRPRDVSENLSP
*M. lucifugus*	XP_006108360	LNPLIKAGIFILKHRRPIGRGIEITGRGIKKFFSK
*M. lucifugus*	XP_006108361	LNPWIIGGALAWKHRRPIGRGLEKAGSGIKRFFSKRSPEQEP
*M. molossus*	XP_036123304	SLGGLLKKGGQIIGKKIEKIGKRIKDFFTNTESMEEAKSV
*M. myotis*	XP_036189569	LNPDTPKPVSFTLKETVCPRTTRQPPEECDFKENGLVKVCGGTVTLDQDTDYYDVHCEEIKDVAIRPLVSGALFLWKNRRPIGWGIEKTGRGIKRIFSKRSPEQEP
*M. myotis*	KAF6310192	AIRPLVSGALFLWKNRRPIGWGIEKTGRGIKRIFSKRSPEQEP
*M. myotis*	KAF6310193	AIRPLVSGALFLWKNRRPIGWGIEKTGRGIKRIFSKRSPEQEP
*M. natalensis*	XP_016058295	KLRGLLGGLLRKGGRKIGEGIEGFGRRIKNFFSNLSPREES
*P. discolor*	KAF6098810	QLGDTEQTAFRGGSTNGEFDRFRRFPPFPRIPRFPRFPRFP
*P. discolor*	KAF6098809	QLGDTEQTAFRGGSTNGEFDRFRRFPPFPRIPRFPRFPRFP
*P. discolor*	XP_035886276	ILGPALRIGGRIAGRIAGKLIGDAINRHRERNRQRRG
*P. discolor*	XP_028374415	ILGPALRIGGRIAGRIAGKLIGDAINRHRERNRQRRG
*P. kuhlii*	XP_036288304	NLDNLIQKGREKLGRLRELFRKGGQKVGKLLQKGGQKLGEIGQRIRDFFSNLRPREEGPQPRGEGPQPPEGGPQLPEEDTQPQEES
*R. aegyptiacus*	XP_036075573	RGLGNLIRRGGRKIGEGIEGLGRRIKGLFSSLESRK
*R. aegyptiacus*	KAF6473429	RGLGNLIRRGGRKIGEGIEGLGRRIKGLFSSLESRK
*R. ferrumequinum*	KAF6312594	KLGGRLREIIRKGGRKIGQGLENIGKRIKDFFSNVQPREES
*R. ferrumequinum*	XP_032988513	KLGGRLREIIRKGGRKIGQGLENIGKRIKDFFSNVQPREES
*R. ferrumequinum*	KAF6312595	SGPFWVAGSDGTWRMELPMEQLTTAPSRAEKAPSTLSSSLNLRPTGTGSAIP
*S. hondurensis*	XP_036905727	GQISKFRRFRNPFRRFRIHGKITVTFR
* *H. sapiens*	NP_004336	LLGDFFRKSKEKIGKEFKRIVQRIKDFLRNLVPRTES

LPS binding domain of CAP18 is highlighted in red. Duplicate peptides are shadowed in gray. * The human cathelicidin peptide LL-37 was included as an internal control of the bioinfomatic analysis.

## Data Availability

No new data were created in this study. Data used for this article are available in the public database (https://www.ncbi.nlm.nih.gov/) accessed on: 10 January 2021.

## Supplementary Materials

The following are available online, Figure S1: S1-Protein sequence of bat cathelicidins in FASTA format.
