# Supplementary material for: Bioinformatic Analysis of Genome-Predicted Bat Cathelicidins
Source: Molecules. 2021 Mar 23;26(6):1811. doi: 10.3390/molecules26061811 (PMC8004601; doi:10.3390/molecules26061811)
Supplement: Supplementary file 1 [file molecules-26-01811-s001.pdf]

Supplementary material

Bioinformatic Analysis and In Vitro Activity of Genome Predicted Bat Cathelicidins

José Manuel Pérez de la Lastra [1,*], Patricia Asensio-Calavia [2], Sergio González-Acosta [1], Victoria Baca-González [1] and Antonio Morales-delaNuez [1]

1. Biotechnology of Macromolecules Research Group, Instituto de Productos Naturales y Agrobiología, (IPNA-CSIC), Avda. Astrofísico Francisco Sánchez, 3, 38206-San Cristóbal de la Laguna, Spain; sergi_glez@hotmail.com (S.G.-A.); victoria@ipna.csic.es (V.B.-G.); morales.delanuez@ipna.csic.es (A.M.-D.)
2. Biological Activity Service, Instituto de Productos Naturales y Agrobiología, (IPNA-CSIC), Avda. Astrofísico Francisco Sánchez, 3, 38206-San Cristóbal de la Laguna, Spain; patriciaac@ipna.csic.es

* Correspondence: jm.perezdelalastra@csic.es

S1-Protein sequence of bat cathelicidins in FASTA format

```
>XP_016058295.1 PREDICTED: cathelicidin antimicrobial peptide [Miniopterus natalensis]
MEAQGASLHGGRWALLLLLLGLATPLATAQVLSQEEAALLAVEEFNRNSSEESLYRLLQLDQQPDGDENP
NTLRPVSFVVKETVCPRRTQLPPEQCDFKENGLMKECVGLTTLDQKKGYFEVNCVELKDVKLRGLLGGLL
RKGGRKIGEGIEGFGRRIKNFFSNLSPREES
>XP_016058242.1 PREDICTED: 15 kDa protein B-like [Miniopterus natalensis]
MAGAWRALVLVVGLAAVACVAQRRRSYEEIVTQALQFFNYGRQGQPLFGLLEAIPVPRSNSTTRTRTLLR
FRIKETVCLSGQRRQPQECAFRDGGEERNCTGALFTLLRTRILTVDCSQDPERQQEVLREKRSAESPEAP
ASDTDISKLPPVARDMYEKAKYDIINNILRNF
>XP_011373748.1 cathelicidin antimicrobial peptide [Pteropus vampyrus]
MARAWMALMLVVGLAATACVAQRRLSYEEIVTEALWIFNQGRRGQSLFGLVEAIPPANSNSTTRTKLSFR
IKETVCIAGRRTEPRECAFKADGVSLPAWASASTHLTWPRGTFREKRSTESAETASAGADTSKLPPAVRD
MYERAKYEIIANILSNF
>EPQ08412.1 Cathelicidin antimicrobial peptide [Myotis brandtii]
METQRNSLWWGRLLLLLLLLGQAMPSPPSPSWALSYQEAVRLAVQGFNQRSREASLYRLLQQDPQPQGDL
NPDTRKPVSFTLKETVCPRTTRQPPEQCDFKKNGVVKECAGTVTLDPDTGYNDVVCEEIKNVELNIENLG
ERIKNAKKKVWEKIKSFGRRIKDFFRKPSPEVEP
>ELK24989.1 Cathelicidin antimicrobial peptide [Myotis davidii]
MEAQRNSLCGGRWPLLLLLLGLAMPWPPAAARALSYQEAVGLAVQGFNQRSREASLYRLLQQDPQPQGDL
NPDTPKPVSFTLKETVCPRTTRQPPEQCDFKENGLVKACAGTVTLDQDTGSYDVVCEGVSGPF
>ELK24988.1 Cathelicidin antimicrobial peptide [Myotis davidii]
MEAQRNSLCGGRWPLLLLLLGLAMGGRTPRLPTARRHRIGRENRARGVDTFWK-
FEMKRRNVRTTRQPPEQCDFKENGLVKVCVGTVTLDQDNGSYDVVCEEIKDVILGAENLGERIKNAKKKVWEKIKSFGRRIKEFFRK
PSPEVEP
>KAF6420041.1 cathelicidin antimicrobial peptide [Molossus molossus]
MKTQRNSLSWGCWSLLLLVVGLAMPPATGQALSYQQAVLLAVQGFNQRSSEANLYRLLELGAKPEGDENP
NTPKPVSFTVKETVCPRTTRQPPEECDFKENGLIKECIGTVDLDPAKGYFDITCDQLKDVSLGGLLKKGG
QIIGKKIEKIGKRIKDFFTNTESMEEAKSV
>KAF6312596.1 cathelicidin antimicrobial peptide [Rhinolophus ferrumequinum]
METQGGSPSLRRWSLLLLLLGLAMPPATAQALTYREAVLRAVDGFNQQSSEASLYRLLELDQQPHGDGNP
NIPQPVSFTVKETVCPRSTQQPTEQCDFKEKGLVKQCVGTVTLDQVDGYFDISCEELRNVKLGGRLREII
RKGGRKIGQGLENIGKRIKDFFSNVQPREES
>KAF6312595.1 cathelicidin antimicrobial peptide [Rhinolophus ferrumequinum]
METQGGSPSLRRWSLLLLLLGLAMPPATAQALTYREAVLRAVDGFNQQSSEASLYRLLELDQQPHGDGNP
NIPQPVSFTVKETVCPRSTQQPTEQCDFKEKGLVKQCVGTVTLDQVDGYFDISCEEVSGPFWVAGSDGTW
RMELPMEQLTTAPSRAEKAPSTLSSSLNLRPTGTGSAIP
>KAF6098810.1 cathelicidin antimicrobial peptide [Phyllostomus discolor]
METQRDNLPWGRWSLLLLLLGLAVPPAAAQVLGYNEAVLAAVDGYNQRSSEANLYRLLELDPQRPDADDN
PNTPKPVSFTVKETVCPRTTQLPPEQCEFQENGLVKQCAGTVSLDQASGYFDINCVEIQDVQLGDTEQTA
FRGGSTNGEFDRFRRFPPFPRIPRFPRFPRFP
>XP_008154130.1 cathelicidin antimicrobial peptide [Eptesicus fuscus]
```

METQRNSLSWGRWSLLLLLLGLAMPMPPTAAQAMSYQEAVRLAVQGFNQRSLEASLYRLLQLEPQPQGDP
NPFTPKPVSFTLKETVCPRTTRRPPEECDFKENGLVKECAGAVTLDPDDGFFDVTCDEIRNVKFNARKLG
ELIRRGGEGFGRKVEKIGRRIKEFFTNLAPREEEA
>XP_005867268.1 PREDICTED: cathelicidin antimicrobial peptide isoform X1 [Myotis brandtii]
METQRNSLWWGRLLLLLLLLGQAMPSPPSPSWALSYQEAVRLAVQGFNQRSREASLYRLLQQDPQPQGDL
NPDTRKPVSFTLKETVCPRTTRQPPEQCDFKKNGVVKECAGTVTLDPDTGYNDVVCEEVSRPF
>XP_036984419.1 cathelicidin antimicrobial peptide [Artibeus jamaicensis]
METQRDSLPWGRWSLWLLLLGLAVPPATAQVVSYNEAVLAAVNGFNQRSSEPNLYRLLELDQQRPDADDN
PDTPKPVSFTVKETVCPRTTQLPPEQCEFKENGVRLGLRAQGACHPKR
>XP_035886276.1 cathelicidin antimicrobial peptide isoform X1 [Phyllostomus discolor]
METQRDNLPWGRWSLLLLLLGLAVPPAAAQVLGYNEAVLAAVDGYNQRSSEANLYRLLELDPQSPDADDN
PNTPKPVSFTVKETVCPRTTQLPPEQCEFQENGLVKQCAGTVSLDQASGYFDINCEEIQDVILGPALRIG
GRIAGRIAGKLIGDAINRHRERNRQRRG
>XP_028374415.1 cathelicidin antimicrobial peptide isoform X2 [Phyllostomus discolor]
METQRDNLPWGRWSLLLLLLGLAVPPAAAQVLSYNQAVLAAVDGFNQRSSEANLYRLLELDPPRPDADDN
PNTPKPVSFTLKETVCPRTTQLPPEQCEFQENGLVKQCAGTVTLGQASSYFDINCAEIQDVILGPALRIG
GRIAGRIAGKLIGDAINRHRERNRQRRG
>KAF6473429.1 cathelicidin antimicrobial peptide [Rousettus aegyptiacus]
MPSLKRWSLLLLLLGLARPPATSAQAFSYQEAVLRAVDGFNQRSKEANLYRLLELDPQLQQEKDKNTPRP
VSFRVKETVCPRTTRQVPEQCDFKENGLVKQCVGTVTLDQVNGYFDINCAELQGVRGLGNLIRRGGRKIG
EGIEGLGRRIKGLFSSLESRK
>KAF6334921.1 cathelicidin antimicrobial peptide [Pipistrellus kuhlii]
METQGSCLCGGRRALLLLLLLGLAMPPPAAQAAQALSYQQAVRLAVLGFNQRSREPSLYRLVRLDPPPPP
GDLSPEARRPVSFTLQETVCPRTTRRPPEECDFREEGLLKECSGSVILDPDNGYFDVTCEEIKKVNLDNL
IQKGREKLGRLRELFRKGGQKVGKLLQKGGQKLGEIGQRIRDFFSNLRPREEGPQPRGEGPQPPEGGPQL
PEEDTQPQEES
>KAF6310193.1 cathelicidin antimicrobial peptide [Myotis myotis]
METQRNNLCWGRWPLLLLLLGLAMPLPPAAAQALSYQEAVRLAVQGFNQRSREASLYRLLQQDPQPQDDL
NPDTPKPVSFTLKETVCPRTTRQPPEECDFKENGLVKVCGGTVTLDQDTDYYDVHCEEIKDVAIRPLVSG
ALFLWKNRRPIGWGIEKTGRGIKRIFSKRSPEQEP
>KAF6310192.1 cathelicidin antimicrobial peptide [Myotis myotis]
MATQRNSLCWGRWPLLLLLLGLAMPLPPAAARALSYQEAVRLAVQGFNQRSREASLYRLLQQDPQPQGDL
NPDTPKPVSFTLKETVCPRTTRQPPEECDFKENGLVKVCGGTVTLDQDTDYYDVHCEEIKDVAIRPLVSG
ALFLWKNRRPIGWGIEKTGRGIKRIFSKRSPEQEP
>XP_024421798.1 cathelicidin antimicrobial peptide isoform X2 [Desmodus rotundus]
MGTQKDSLPWRRWSLLLLLLVLVVPPATAQVLSYNEAVLRAIEDLNQRSSEASLYRLLGLDQQPPDGDEN
PNTPKPVSFTVKETVCPRTTQLPPEQCEFKENGLVKQCTGTVTLAQANDSFDINCADIQDVRVPGWLRKT
GRAIGNAIRIVGPILPIFFPRG
>XP_024421797.1 cathelicidin antimicrobial peptide isoform X1 [Desmodus rotundus]
MGTQKDSLPWRRWSLLLLLLVLVVPPATAQVLSYNEAVLRAIEDLNQRSSEASLYRLLGLDQQPPDGDEN
PNTPKPVSFTVKETVCPRTTQLPPEQCEFKENGLVKQCTGTVTLAQANDSFDINCADIQDVGIRSGVQRI
VDKIRDIGRRINDFFSNLFPRGVS
>XP_019486615.1 PREDICTED: cathelicidin antimicrobial peptide [Hipposideros armiger]
METQSGSPSLKRWSLLLLLLGLAIPPATAQALTYREAVLRAVDGFNQRSSEASLYRLLELDPLPGGDDNP
NIPKPVSFTVKETVCPRRTPQLETTRRPTEQCAFKEKGLVKQCVGTVTLDQEDGYFDITCEKLQNVILGR
LRDLLRRGGRKIGQGLERIGQRIQGFFSNREPMEES
>EPQ08403.1 15 kDa protein A [Myotis brandtii]
MACEAQYRLGTQERIALQALHFFNAGRQGQPEFGLVEVLPTARSNSTLRTLVRFRIKETVCLSGRQQQPP
QQCAFRDGGEERNCTSAFRRRSFLSRLSILLVDCSPGPERQQEDSREQPTAPPPEEEAASDIDRSKLPPV
VRGLYDNARNDIINNILRNF
>ELK10182.1 hypothetical protein PAL_GLEAN10007854 [Pteropus alecto]
MARAWMALMLVVGLAATACVAQRHLSYEEIVTEALRIFNQGRWGQSLFGLVEAIPPAN-
SEERNCTGIFFMGHGRILLVDCRPGPLRQPETFREKRSTESAKTASAGADTSKLPPAVRDMYERAKYDIIANILSNF
>XP_008156033.1 15 kDa protein A-like [Eptesicus fuscus]
MAGAWRALVLAAGLAAMACVAQRQWGFEERVVAQALQLFNSGRQGQPLSRLREVLPAPRSNSTFKTLVKF
RVKETVCLSERQQQPEPQQCAFRDGGEERDCTAASFTRGPLHILMVDCNPGPERQQEGLREKRSAPSPEG
AASEIDSSQLPPVARDLYEKAKFDLIANLLRNF
>XP_014306451.1 15 kDa protein B-like [Myotis lucifugus]
MAGAWRALVLVAGLAAMACEAQHRRFPPERIAAQALQFFNSERQGQPLFGLLEVLPTARSNSTLRTLVRF
RIKETVCLSGRQQELEPQQCAFRDGGEERNCTSTFGRRGRLSILLVDCSPGPERQQEDSREQPTAPAPPP
EEAASDIDRSKLPPAVRDLYDNARNNIINNILRNF
>XP_006108362.1 cathelin-like [Myotis lucifugus]

```
METQRSRLCWGCWPLLLLLLGLAIPTPLAAARALSYQEAVRLAVQGFNQRSREASLYRLLQQDPQPQGDL
NPDTPKPVSFTVKETVCPRTTRQPPEECDFKENGLVKECAGTVTLDQDTGYYDVVCEEIKDVRFNYDRLS
NIIKRGGYKLGEGLEIVGGILRRS
>XP_006108361.1 cathelin-like [Myotis lucifugus]
METQRNSLCWGHWSLLLLLLGLAMPTPPAAAWALSYQEAVRLVVQGFNQRSREASLYRLLQQDPQPQGDL
NPDTPKPVSFTVKETVCPRTTRQPPEQCDFKENGLVKECAGTVTLDPDNGYIDVVCEEIKNVLNPWIIGG
ALAWKHRRPIGRGLEKAGSGIKRFFSKRSPEQEP
>XP_005867266.1 PREDICTED: 15 kDa protein B [Myotis brandtii]
MAGAWRALVLVAGLAAMACEAQYRLGTQERIALQALHFFNAGRQGQPEFGLVEVLPTARSNSTLRTLVRF
RIKETVCLSGRQQQPPQQCAFRDGGEERNCTSAFRRRSFLSRLSILLVDCSPGPERQQEDSREQPTAPPP
EEEAASDIDRSKLPPVVRGLYDNARNDIINNILRNF
>XP_006770630.2 PREDICTED: 15 kDa protein B-like [Myotis davidii]
MAGAWRALVLVAGLAAMACEAQHRPDPQAKIVAQALRFFNAGRQGQPLFGLLEVLPTPRSNSTLRTLVRF
RIKETVCLSERQQQPAPQQCAFRDGGEERTCTSAFGRLGRQRFLLVDCSPGPERQQEGSPEQPEQPPAPP
PEEAASDIDRSKLPPVARNLYDNARNDIINNLLKNF
```
